# Associations between eating alone, its transition and depressive symptoms among Chinese middle-aged and older adults: evidence from two national cohorts

**DOI:** 10.1186/s12888-024-05909-7

**Published:** 2024-06-19

**Authors:** Baohua Qiu, Rongyu Zhu, Xinlu Huang, Zhijuan Qi, Lijuan Zhang

**Affiliations:** 1 Shiquan Community Health Service Center, Tongji University School of Medicine, Shanghai, China; 2 Shanghai Putuo District Health Affairs Management Center, Shanghai, China; 3 Clinical Center for Intelligent Rehabilitation Research, Shanghai YangZhi Rehabilitation Hospital (Shanghai Sunshine Rehabilitation Center), Tongji University School of Medicine, Shanghai, China; 4 School of Public Health and General Practice, Tongji University School of Medicine, Tongji University, Shanghai, China

**Keywords:** Eating alone, Depressive symptoms, Middle-aged and older adults, Cohort study, China

## Abstract

**Background:**

Few studies have explored the longitudinal association between eating alone and depressive symptoms, and have focused on the effect of eating alone transition. This study aims to explore the longitudinal association between eating alone, its transition and depressive symptoms among two national surveys using a cohort study design.

**Methods:**

The participants aged ≥ 45 years were recruited for the 2016 to 2018 waves China Family Panel Data (CFPS) and 2015 to 2018 waves China Health and Retirement Longitudinal Study (CHARLS). Eating alone was assessed by self-reported. Depressive symptoms were evaluated by Center for Epidemiologic Studies Depression Scale. Cox hazard regression was used to explore the associations between eating alone, its transition and depressive symptoms after adjusting for covariates.

**Results:**

A total of 21,476 participants were included in this study. The Cox model showed that compared with commensality, eating alone was associated with a higher risk of depressive symptoms, both in the CFPS, CHARLS and pooled analysis. In addition, compared with commensality consistently, the transition from commensality to alone and eating alone consistently were associated with a higher risk of depressive symptoms. The sensitivity analyses showed that the association remained robust.

**Conclusions:**

Eating alone and a change from commensality to eating alone were associated with higher risks of depressive symptoms among Chinese middle-aged and older adults in two cohorts. This study suggested that providing eating partners may be an effective intervention method to prevent depressive symptoms in middle-aged and older adults.

**Supplementary Information:**

The online version contains supplementary material available at 10.1186/s12888-024-05909-7.

## Background

Depressive symptoms among middle-aged and older people are a major challenge facing current society, especially in low- and middle-income countries (LMICs). A previous national survey in LMICs showed that the prevalence of depression symptoms among middle-aged and older people aged ≥ 45 years in India, China, and Thailand was 27.6% [1], 36.7% [2] and 19.4% [3], respectively. However, the prevalence of depressive symptoms in high-income countries such as the UK and the US was 13.6% [4] and 8.7% [5], respectively. Due to differences in health resources and economic levels, LMICs faced a more challenging situation in the prevention and control of depressive symptoms than high-income countries. Previous studies have demonstrated that depressive symptoms were associated with multiple adverse health outcomes in LMICs, such as pain [6], higher suicide attempts [7], dementia [8], cardiovascular disease and all-cause mortality [9]. In addition, depressive symptoms in middle-aged and older adults were also associated with higher health service utilization and catastrophic medical expenditures [10], which can additionally increase the national healthcare burden and the economic burden on families. In view of the high prevalence, health hazards and disease burden of depressive symptoms, it is necessary to further clarify its related risk factors and conduct early prevention and intervention plans based on these factors, which is essential for the well-being of middle-aged and older adults in LMICs.

Previous systematic reviews and meta-analyses have explored risk factors for depressive symptoms, such as demographic factors (such as age and sex), socioeconomic status, behavior and lifestyle (such as smoking and drinking), and psychological and social factors (such as loneliness and social support) [11, 12]. In addition, the social determinants of health considered the social environment and social relationships as important factors influencing the health of individuals [13]. A recent scoping review also clarified that social connectedness and social ties is a determinant of individual mental health, such as depressive symptoms [14]. Diet is a necessary daily necessity for everyone, and eating companionship is considered an important part of an individual’s social relationships [15]. A cross-sectional survey of Chinese people aged ≥ 60 years showed that the proportion of people eating alone was 17.1% [16]. A Japanese survey showed that 15.5% of 10,308 older people aged 65 to 84 ate alone [17]. In addition, the Korea National Health and Nutrition Examination Survey (KNHANES) showed that the rate of eating alone was 21.8% in 7,278 Korean older adults aged ≥ 65 years [18]. As a sign of social disengagement, eating alone has been associated with a variety of physical and mental health problems [19]. A Korean cross-sectional study found that eating alone is associated with a higher prevalence of metabolic syndrome among adults with a mean age of 47.1 years [20]. A five-year longitudinal study in Japan suggested that eating alone is associated with an increased risk of functional impairment among community-dwelling older adults aged 65–84 years [17]. A Japanese cross-sectional study also found that eating alone was associated with poor appetite among older people aged 70 and over [21]. Moreover, the association between eating alone and depressive symptoms has also been confirmed in multiple studies. For example, a cross-sectional study based on KNHANES suggested that Korean adults aged ≥ 65 years who ate alone had a higher rate of depressive symptoms than those who ate together [22]. The Furukawa nutrition and health study also found that a lower frequency of commensality is associated with a higher prevalence of depressive symptoms among Japanese workers with a mean age of 45.4 years [23]. A cross-sectional study of older adults in China also showed that eating alone is a strong risk factor associated with depressive symptoms among older adults aged 60 years and above [16]. However, the direction of the association between eating alone and depressive symptoms remains unclear because cross-sectional studies cannot infer the temporal order between variables. To our knowledge, only one Japanese cohort study has explored the longitudinal relationship between eating alone and depressive symptoms [24], but its applicability to China is still unknown. Meanwhile, it is important to note that eating alone is not set in stone and may change over time. However, to our knowledge, no studies have focused on the association between the transition of eating alone and depressive symptoms.

Therefore, to fill the gap in the literature and minimize associations by chance, this study aims to assess the longitudinal associations between eating alone, its transition and depressive symptoms among Chinese middle-aged and older adults based on two national cohorts with a larger sample size.

## Methods

### Participants

**Fig. 1:**
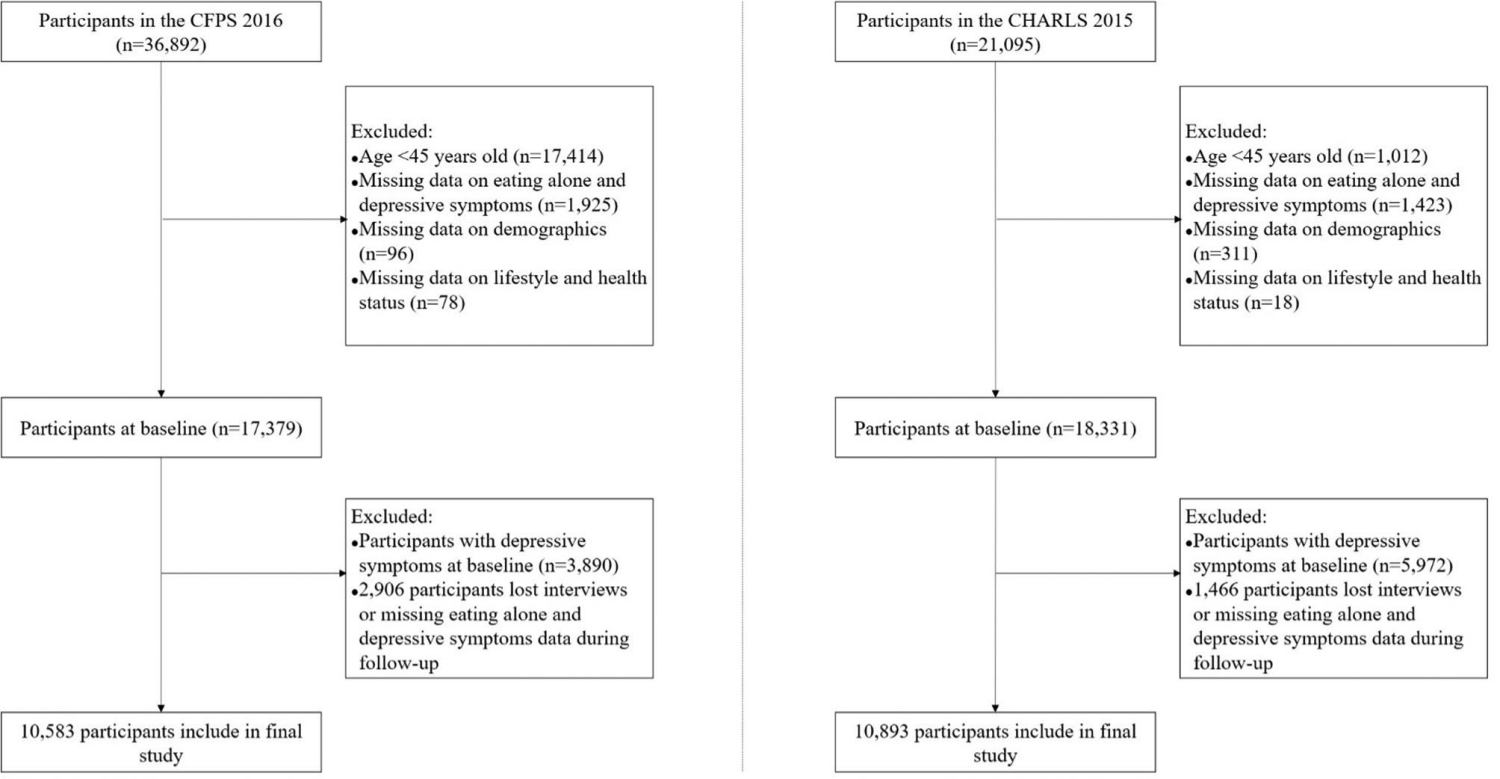
The selection process of the participants

The participants in this study were recruited from the 2016 to 2018 waves of the China Family Panel Studies (CFPS) and the 2015 to 2018 waves of the China Health and Retirement Longitudinal Study (CHARLS). Both CFPS and CHARLS are national longitudinal survey projects implemented by Peking University using the multimethod of probability proportional to size sampling. CFPS survey sites cover 25 provincial administrative units in China and have followed approximately 16,000 households nationwide since 2010, with all members of the household included in the survey; details of implementation have been reported in a previous study [25]. CHARLS is a nationwide follow-up survey of Chinese middle-aged and older people aged 45 and above conducted since 2011, covering 28 provincial administrative regions and including more than 20,000 participants, with good national representativeness. A previous study provided detailed information on the study design, sample recruitment, and data collection of the CHARLS [26]. In this study, we selected data from the 2016 to 2018 waves of the CFPS and 2015 to 2018 waves of the CHARLS for analysis. Inclusion criteria for study participants were age ≥ 45 years and no missing information at baseline; exclusion criteria were the presence of depressive symptoms at baseline, and missing data on eating alone and depressive symptoms at follow-up. Individuals who had died, lost their cases, or ended their follow-up were also excluded. Finally, a total of 21,476 middle-aged and older adults (10,583 in CFPS and 10,893 in CHARLS) were included in this cohort studies. All investigations were conducted by uniformly trained investigators at the participants’ homes. The selection process of participants in this study is displayed in Fig. 1. Both the CFPS (ethical approval code: IRB00001052-14010) and CHARLS (ethical approval code: IRB00001052-11015) have been approved by the Biomedical Ethics Committee of Peking University, and all participants signed an informed consent form.

### Measurements

#### Eating alone

The assessment of eating alone was similar in the CFPS and CHARLS, both of which asked participants “In the past week, how many people usually ate meals in your household?” Because older people have been at home for the past week, if the participant answered one person, it was considered to be eating alone; otherwise, they were eating with others. Based on changes at baseline and follow-up, we divided the individual feeding conversions into four groups: commensality consistently, from eating alone to with others, from commensality to alone, and eating alone consistently.

#### Depressive symptoms

Depressive symptoms were evaluated by the Center for Epidemiologic Studies Depression Scale (CESD). The difference was that the CFPS uses the 8-item version of the CESD, while the CHARLS uses the 10-item version. All items asked participants about their sensory and behavioral status in the past week, and the answers included < 1 day (0 points), 1–2 days (1 point), 3–4 days (2 points), and 5–7 days (3 points). The total CESD score was obtained by summing all items, with higher scores indicating more severe depressive symptoms. Due to the different versions of CESD used by the two cohorts, the CESD score of ≥ 9 on the CFPS [27, 28] and ≥ 10 on the CHARLS [29] was identified as the presence of clinically significant depressive symptoms.

#### Covariate

Based on the factors related to depressive symptoms found in previous studies [30, 31], we selected age, sex (male vs. female), residence (urban vs. rural), marital status (married vs. unmarried), educational level (illiteracy, primary school vs. middle school and above), family income (≤ the median is low, and > the median is high), smoking (no vs. yes), drinking (no vs. yes), exercise (no vs. yes), health insurance (no vs. yes), pension (no vs. yes), and chronic disease (no vs. yes) as covariates. Among them, the original categories for marital status included never married, married, divorced, and widowed. In this study, we consolidated the never married, divorced, and widowed categories into a single group labeled as unmarried. Exercise was evaluated by asking participants if they have engaged in physical exercise in the past week, and the answer included yes and no.

### Statistical analysis

We used mean (M) ± standard deviation (SD) to describe continuous variables and frequency (composition ratio) to describe categorical variables. We used Cox hazard regression to explore the associations between eating alone, its transition and depressive symptoms. Specifically, we conducted three models: Model 1 was an unadjusted model; Model 2 adjusted sociodemographic characteristics, such as age, sex, residence, marital status, educational level, and family income; and Model 3 further adjusted for smoking, drinking, exercise, health insurance, pension, and chronic disease based on Model 2. The hazard ratio (HR) and 95% confidence interval (CI) were used to indicate the strength of the association. To summarize the associations between eating alone, its transition and depressive symptoms across the two cohorts, HRs from Cox models were pooled across the two cohorts using fixed-effect meta-analysis. Q-statistics were also computed to determine heterogeneity among the two cohorts. Moreover, we analyzed the interaction between eating alone, its transition and covariates and conducted subgroup analyses to understand the potential moderating role of different covariates. In observational epidemiological studies, it is a common practice to include covariates associated with both exposure and outcome in the regression model to conduct sensitivity analysis [32]. Prior research has indicated that individuals with low life satisfaction and the presence of social isolation are more prone to experience eating alone and depressive symptoms [19, 33]. This suggests that life satisfaction and social isolation may be significant confounding factors that influence the relationship between eating alone and depressive symptoms. To test the robustness of this association between eating alone, we further incorporated two covariates, life satisfaction and social isolation in Cox regression analysis. Among these, life satisfaction was self-rated as satisfactory, moderate and unsatisfactory. Social isolation was assessed based on an index of social isolation ranging from 0 to 4 points previously validated in older Chinese adults [34], which included being unmarried (0 = no, 1 = yes), living alone (0 = no, 1 = yes), having contact with parents or children less than once a week (0 = no, 1 = yes), and not participating in social activities during the past month (0 = no, 1 = yes). A score greater than or equal to 2 was judged to indicate social isolation. To avoid the effect of heteroscedasticity, we estimated robust standard error (SE) in the Cox model to eliminate potential bias [35]. We performed all analyses using STATA software (version 17.0), and *P* < 0.05 indicated statistical significance.

## Results

### Descriptive statistics

The characteristics of the participants according to depressive symptoms are displayed in Table 1. The mean follow-up for the CFPS and CHARLS were two and three years, respectively. The mean ages of the participants in the CFPS and CHARLS were 58.2 ± 9.3 and 59.4 ± 9.7 at baseline, respectively. The rates of eating alone in the CFPS and CHARLS were 5.0% and 7.4%, respectively. In addition, the incidences of depressive symptoms at follow-up in the CFPS and CHARLS were 15.7% and 23.4%, respectively. More details are provided in Table 1.

**Table 1:** Characteristics of participants

Variable	CFPS			CHARLS		
	Total (*N* = 10,583)	Depressive symptoms		Total (*N* = 10,893)	Depressive symptoms	
		No (*N* = 8,917)	Yes (*N* = 1,666)		No (*N* = 8,345)	Yes (*N* = 2,548)
Age (years)						
45–59	6076 (57.4)	5151 (57.8)	925 (55.5)	5706 (52.4)	4357 (52.2)	1349 (52.9)
≥ 60	4507 (42.6)	3766 (42.2)	741 (44.5)	5187 (47.6)	3988 (47.8)	1199 (47.1)
Sex						
Male	5579 (52.7)	4871 (54.6)	708 (42.5)	5797 (53.2)	4656 (55.8)	1141 (44.8)
Female	5004 (47.3)	4046 (45.4)	958 (57.5)	5096 (46.8)	3689 (44.2)	1407 (55.2)
Residence						
Urban residents	5098 (48.2)	4470 (50.1)	628 (37.7)	3465 (31.8)	2802 (33.6)	663 (26.0)
Rural residents	5485 (51.8)	4447 (49.9)	1038 (62.3)	7428 (68.2)	5543 (66.4)	1885 (74.0)
Marital status						
Married	9668 (91.4)	8202 (92.0)	1466 (88.0)	9822 (90.2)	7542 (90.4)	2280 (89.5)
Unmarried	915 (8.6)	715 (8.0)	200 (12.0)	1071 (9.8)	803 (9.6)	268 (10.5)
Educational level						
Illiteracy	3014 (28.5)	2358 (26.4)	656 (39.4)	2271 (20.8)	1682 (20.2)	589 (23.1)
Primary school	2566 (24.2)	2145 (24.1)	421 (25.3)	4086 (37.5)	2988 (35.8)	1098 (43.1)
Middle school and above	5003 (47.3)	4414 (49.5)	589 (35.4)	4536 (41.6)	3675 (44.0)	861 (33.8)
Income						
Low	5512 (52.1)	4445 (49.8)	1067 (64.0)	5450 (50.0)	4141 (49.6)	1309 (51.4)
High	5071 (47.9)	4472 (50.2)	599 (36.0)	5443 (50.0)	4204 (50.4)	1239 (48.6)
Smoking						
No	7338 (69.3)	6120 (68.6)	1218 (73.1)	7275 (66.8)	5526 (66.2)	1749 (68.6)
Yes	3245 (30.7)	2797 (31.4)	448 (26.9)	3618 (33.2)	2819 (33.8)	799 (31.4)
Drinking						
No	8516 (80.5)	7103 (79.7)	1413 (84.8)	6629 (60.9)	4947 (59.3)	1682 (66.0)
Yes	2067 (19.5)	1814 (20.3)	253 (15.2)	4264 (39.1)	3398 (40.7)	866 (34.0)
Exercise						
No	5912 (55.9)	4850 (54.4)	1062 (63.7)	8593 (78.9)	6516 (78.1)	2077 (81.5)
Yes	4671 (44.1)	4067 (45.6)	604 (36.3)	2300 (21.1)	1829 (21.9)	471 (18.5)
Health insurance						
No	637 (6.0)	523 (5.9)	114 (6.8)	842 (7.7)	611 (7.3)	231 (9.1)
Yes	9946 (94.0)	8394 (94.1)	1552 (93.2)	10,051 (92.3)	7734 (92.7)	2317 (90.9)
Pension						
No	1968 (18.6)	1608 (18.0)	360 (21.6)	2616 (24.0)	1963 (23.5)	653 (25.6)
Yes	8615 (81.4)	7309 (82.0)	1306 (78.4)	8277 (76.0)	6382 (76.5)	1895 (74.4)
Chronic disease						
No	8413 (79.5)	7200 (80.7)	1213 (72.8)	4793 (44.0)	3861 (46.3)	932 (36.6)
Yes	2170 (20.5)	1717 (19.3)	453 (27.2)	6100 (56.0)	4484 (53.7)	1616 (63.4)
Eating alone						
No	10,052 (95.0)	8503 (95.4)	1549 (93.0)	10,083 (92.6)	7752 (92.9)	2331 (91.5)
Yes	531 (5.0)	414 (4.6)	117 (7.0)	810 (7.4)	593 (7.1)	217 (8.5)
The eating alone transition						
Commensality consistently	9,766 (92.3)	8,281 (92.9)	1,485 (89.1)	9505 (87.3)	7341 (88.0)	2164 (84.9)
From eating alone to commensality	234 (2.2)	184 (2.1)	50 (3.0)	400 (3.7)	301 (3.6)	99 (3.9)
From commensality to alone	286 (2.7)	222 (2.5)	64 (3.8)	578 (5.3)	411 (4.9)	167 (6.6)
Eating alone consistently	297 (2.8)	230 (2.6)	67 (4.0)	410 (3.8)	292 (3.5)	118 (4.6)

### Association between eating alone and depressive symptoms

The results of the association between eating alone and depressive symptoms are displayed in Table 2. In all models, a significant association between eating alone and depressive symptoms was consistently found in the CFPS and CHARLS. In the unadjusted model, eating alone was significantly associated with a higher risk of depressive symptoms in the CFPS (HR = 1.430, 95% CI = 1.211–1.689) and CHARLS (HR = 1.159, 95% CI = 1.029–1.306). After adjusting for sociodemographic characteristics, eating alone was also still associated with depressive symptoms in the CFPS (HR = 1.370, 95% CI = 1.143–1.641) and CHARLS (HR = 1.162, 95% CI = 1.019–1.324). In the fully adjusted model, compared with noneating alone, eating alone was still associated with a higher risk of depressive symptoms, both in the CFPS (HR = 1.369, 95% CI = 1.142–1.640) and CHARLS (HR = 1.168, 95% CI = 1.026–1.329). In our pooled meta-analysis, we also found a significant association between eating alone and depressive symptoms (HR = 1.232, 95% CI = 1.109–1.369, Q = 1.96, *P*_heterogeneity_ = 0.162).

**Table 2:** Associations between eating alone and depressive symptoms

Model		CFPS				CHARLS		
	HR (95%CI)		Robust SE	*P*	HR (95%CI)		Robust SE	*P*
Model 1								
Non-eating alone	Reference				Reference			
Eating alone	1.430 (1.211–1.689)		0.121	< 0.001	1.159 (1.029–1.306)		0.071	0.015
Model 2								
Non-eating alone	Reference				Reference			
Eating alone	1.370 (1.143–1.641)		0.126	0.001	1.162 (1.019–1.324)		0.078	0.025
Model 3								
Non-eating alone	Reference				Reference			
Eating alone	1.369 (1.142–1.640)		0.126	0.001	1.168 (1.026–1.329)		0.077	0.019

### Association between eating alone transition and depressive symptoms

The results of the association between eating alone transition and depressive symptoms are displayed in Table 3. In the three models of CFPS, compared with commensality consistently, the transitions from eating alone to commensality, from commensality to eating alone, and eating alone consistently were all significantly associated with a higher incidence of depressive symptoms. However, in the CHARLS, the association between the transition from eating alone to commensality and depressive symptoms was not significant. Compared with commensality consistently, the transition from commensality to alone and eating alone consistently were significantly associated with a higher incidence of depressive symptoms in CHARLS. In pooled analysis, the transitions from eating alone to commensality (HR = 1.221, 95% CI = 1.058–1.408, Q = 3.12, *P*_heterogeneity_ = 0.077), from commensality to eating alone (HR = 1.294, 95% CI = 1.156–1.448, Q = 0.43, *P*_heterogeneity_ = 0.513), and eating alone consistently (HR = 1.314, 95% CI = 1.141–1.514, Q = 0.02, *P*_heterogeneity_ = 0.884) were all significantly associated with a higher incidence of depressive symptoms.

**Table 3:** Associations between eating alone transition and depressive symptoms

Model		CFPS				CHARLS		
	HR (95%CI)		Robust SE	*P*	HR (95%CI)		Robust SE	*P*
Model 1								
Commensality consistently	Reference				Reference			
From eating alone to commensality	1.405 (1.094–1.805)		0.179	0.008	1.087 (0.913–1.295)		0.097	0.349
From commensality to alone	1.472 (1.180–1.835)		0.166	0.001	1.269 (1.111–1.450)		0.086	< 0.001
Eating alone consistently	1.484 (1.196–1.841)		0.163	< 0.001	1.264 (1.081–1.479)		0.101	0.003
Model 2								
Commensality consistently	Reference				Reference			
From eating alone to commensality	1.487 (1.155–1.915)		0.192	0.002	1.121 (0.940–1.337)		0.101	0.203
From commensality to alone	1.369 (1.099–1.705)		0.153	0.005	1.261 (1.104–1.441)		0.086	0.001
Eating alone consistently	1.325 (1.045–1.680)		0.160	0.020	1.284 (1.075–1.533)		0.116	0.006
Model 3								
Commensality consistently	Reference				Reference			
From eating alone to commensality	1.473 (1.144–1.897)		0.190	0.003	1.117 (0.939–1.329)		0.099	0.212
From commensality to alone	1.378 (1.106–1.718)		0.155	0.004	1.265 (1.109–1.442)		0.085	< 0.001
Eating alone consistently	1.333 (1.050–1.691)		0.162	0.018	1.304 (1.093–1.555)		0.117	0.003

Notes: Model 1 was unadjusted model; model 2 adjusted for age, sex, residence, marital status, education level, and income; model 3 further adjusted for smoking, drinking, exercise, health insurance, pension, and chronic disease based on model 2

### Subgroup analysis

The results of subgroup analysis in CFPS and CHARLS are displayed in Supplementary materials. Although there are slight differences in the associations between eating alone, its transition and depressive symptoms among the subgroups, the interactions are all not significant.

### Sensitivity analysis

Table 4 shows the results of sensitivity analyses. The results suggested that after further adjusting for life satisfaction and social isolation, eating alone was still associated with a higher incidence of depressive symptoms in the CFPS and CHARLS. In addition, the association between eating alone transition and depressive symptoms in the CFPS and CHARLS was also consistent with the results of model 3. In pooled analysis, eating alone (HR = 1.194, 95% CI = 1.080–1.320, Q = 2.13, *P*_heterogeneity_ = 0.144) was also associated with a higher incidence of depressive symptoms. In addition, compared with commensality consistently, the transitions from eating alone to commensality (HR = 1.194, 95% CI = 1.037–1.376, Q = 3.85, *P*_heterogeneity_ = 0.050), from commensality to eating alone (HR = 1.281, 95% CI = 1.145–1.434, Q = 0.52, *P*_heterogeneity_ = 0.472), and eating alone consistently (HR = 1.242, 95% CI = 1.087–1.420, Q = 0.08, *P*_heterogeneity_ = 0.777) were all significantly associated with depressive symptoms. These results showed that the associations between eating alone, its transition and depressive symptoms were robust.

**Table 4:** Sensitivity analyses

Sensitivity analyses		CFPS				CHARLS		
	HR (95%CI)		Robust SE	*P*	HR (95%CI)		Robust SE	*P*
Eating alone								
Non-eating alone	Reference				Reference			
Eating alone	1.337 (1.114–1.604)		0.124	0.002	1.136 (1.007–1.282)		0.070	0.038
Eating alone transition								
Commensality consistently	Reference				Reference			
From eating alone to commensality	1.475 (1.144–1.901)		0.191	0.003	1.086 (0.916–1.288)		0.094	0.343
From commensality to alone	1.373 (1.102–1.709)		0.154	0.005	1.250 (1.096–1.424)		0.083	0.001
Eating alone consistently	1.278 (1.008–1.620)		0.155	0.043	1.226 (1.043–1.442)		0.101	0.014

## Discussion

In this study, we used two national cohorts to explore the longitudinal association between eating alone, its transition, and depressive symptoms among Chinese middle-aged and older adults. To our knowledge, this is the first study to explore the above association, which provides higher-level research evidence for the prevention and intervention of depression symptoms in middle-aged and older people from the perspective of eating companionship in the future. In both the respective and pooled analyses of the two cohorts, we found that eating alone is associated with an increased risk of depressive symptoms among Chinese middle-aged and older adults. In addition, compared with participants who consistently exhibited commensality, the transition from commensality to alone and eating alone consistently were associated with a higher risk of depressive symptoms.

Although a previous cross-sectional study has explored the relationship between eating alone and depressive symptoms [16], its participants were only selected in Zhejiang Province (one of the 34 provincial administrative regions in China), which means that the extrapolation of this study is poor. Our study further explored the association using two large nationally representative cohorts, which allowed us to generalize our findings to the entire Chinese middle-aged and older population. In addition, we used a cohort study design that yielded higher causal ranks for the association of eating alone with depressive symptoms than the cross-sectional study [16]. The mechanism of association between eating alone and depressive symptoms may involve two aspects: social relation limitation and nutritional status. On the one hand, in China, people often prefer to eat with family or friends, as eating is considered a social move, and people tend to share food and communicate about each other’s lives or enhance established relationships at the dinner Table [36]. Middle-aged and older adults who eat alone may not have access to social interaction and social participation through the dinner table, leading to social relation limitations. A previous systematic review also suggested that social relations are a strong influencing factor for depression in late life [37]. Moreover, social relation limitations can also induce loneliness, which can lead to depressive symptoms. For example, a cross-sectional survey in China found that intergenerational relationships affected depressive symptoms although loneliness among older adults [38]. On the other hand, in China, the newest dietary guidelines for older Chinese adults included one of four principles to encourage eating together, maintaining a good appetite, and enjoying good food [39]. Eating alone may affect the appetite of middle-aged and older people [21], thus reducing their food intake and leading to an increased risk of malnutrition [40]. A previous study based on the CHARLS confirmed that malnutrition is significantly associated with depressive symptoms [41]. These studies provide support for our interpretation of the association between eating alone and depressive symptoms.

In addition, we also explored the association between eating alone transition and depressive symptoms. A previous Korean cohort study also explored the association between eating alone transition and frailty in older adults and found that the shift from commensality at baseline to eating alone at follow-up was associated with a higher risk of worsening frailty [42]. A meta-analysis of 8,023 older adults in 24 study designs showed that frail older adults were significantly more likely to suffer from depressive symptoms [43], which indirectly supports our finding that a shift from commensality to eating alone was associated with a higher risk of depressive symptoms. In addition, another 3-year prospective study in Korea also found that among older women, the greatest decline in cognitive function was seen in the group moving from commensality to eating alone compared to the other three groups (commensality consistently, from eating alone to commensality, and eating alone consistently) [44]. Similarly, a previous systematic review and meta-analysis involving 120,000 participants suggested that low cognitive function increases the risk of subsequent depressive symptoms [45]. The shift from commensality to eating alone is actually a sign of shrinking social relationships and social networks in middle-aged and older adults, which can easily lead to social isolation and thus increase the risk of depressive symptoms [46]. Meanwhile, previous studies also found that individuals with social isolation (such as living alone) have a high rate of eating alone [47, 48]. Based on the above studies, we hypothesized that social isolation may be a confounder between the association of eating alone and depressive symptoms, and therefore, we performed sensitivity analysis with social isolation as a covariate. However, eating alone and the shift from commensality to eating alone remained associated with depressive symptoms in sensitivity analyses, which suggests that eating alone is a risk factor for depressive symptoms in middle-aged and older adults independent of social isolation. Based on the findings of this study, we suggest that centralized dining environments should be established for middle-aged and older adults, such as group kitchens and dining rooms in communities or villages, so that people who live alone or do not have eating partners can eat together, which may help reduce their depressive symptoms. It is crucial to acknowledge that this study did not observe a decrease in the risk of depressive symptoms associated with the transition from eating alone to commensality; rather, it identified an increase in such risk. We speculate that participants accustomed to eating alone may experience heightened anxiety and stress when adapting to the social dynamics of commensality. This transition might exacerbate perceived interpersonal conflicts, including generational differences with family members, value clashes, or lifestyle discrepancies. These tensions could contribute to an escalation in depressive symptoms. As participants’ eating companions and the reason of transition were not investigated in two cohorts, which limits our discussion. Therefore, further comprehensive analysis exploring the role of eating companionship and reasons for the transition in the relationship between the transition from eating alone to commensality and depressive symptoms.

This study has the following three advantages in terms of sample representativeness, scientific design and statistical analysis. First, we explored the association between eating alone, its transition and depressive symptoms based on two nationally representative large sample cohorts, and the findings are highly extrapolative and can be used to guide the development of depressive symptom prevention and intervention strategies for middle-aged and older adults in China as a whole. Second, we explored the above associations for the first time using a prospective cohort study design in China, which has a higher level of evidence based on the perspective of evidence-based medicine [49]. Third, we controlled for a large number of covariates in the Cox model and included life satisfaction and social isolation in the sensitivity analysis, which suggests that our findings are robust.

In addition, our study has some limitations. First, given that observational studies cannot infer causality, although we used a cohort study to explore the association between eating alone and depressive symptoms, this was not causal. Future randomized controlled trials could be used to explore differences in depressive symptoms in older adults in the eating alone and commensality groups. Second, we selected only two waves of the CFPS and CHARLS for the study, with a short follow-up period of two (CFPS) or three years (CHARLS), and it is necessary to further extend the follow-up period in the future to observe the long-term associations of the above variables. Third, during the sample selection process, we excluded some participants due to missing data or missing interviews, which may introduce selection bias. Finally, although we included many covariates in the Cox model, there are undeniably some covariates associated with eating alone and depressive symptoms that were not included, such as genetic predisposition and environmental factors, which may also produce confounding bias. Furthermore, due to the fact that neither the CFPS nor the CHARLS investigated specific dietary and food information, we are unable to control for dietary factors in our model, such as diet quality and dietary pattern. Unhealthy diet (proinflammatory dietary pattern) has been associated with greater depressive symptomatology in previous evidence [50]. In turn, interventions based on diet and the social component of diet (e.g. Mediterranean diet-based interventions) could have a positive impact on depressive symptoms [51]. Future research should incorporate additional dietary covariates and analyze their interactions with eating alone to elucidate the relationships between dietary companionship, dietary quality, dietary patterns, and depressive symptoms more clearly. Finally, this study also conducted subgroup and interaction analysis. Unfortunately, although there were differences in some subgroups, no significant interaction was found. The differences between these subgroups may be due to a decrease in sample size after grouping, and further exploration of subgroup results and interactions is needed in larger sample studies in the future.

## Conclusions

Eating alone and a change from commensality to eating alone were associated with higher risks of depressive symptoms among Chinese middle-aged and older adults in two cohorts. This study suggested that providing eating partners may be an effective intervention method to prevent depressive symptoms in middle-aged and older adults.

### Electronic supplementary material

Below is the link to the electronic supplementary material.

Supplementary Material 1

## Data Availability

The data of this study can be obtained on the official website of CFPS (http://www.isss.pku.edu.cn/cfps/) and CHARLS (http://charls.pku.edu.cn/).

## Abbreviations

CFPS: China Family Panel Data()
CHARLS: China Health and Retirement Longitudinal Study
LMICs: Low- and middle-income countries
KNHANES: Korea National Health and Nutrition Examination Survey
CESD: Center for Epidemiologic Studies Depression Scale
SD: Standard deviation
CI: Confidence interval
SE: Standard error
