# Supplementary material for: Associations between eating alone, its transition and depressive symptoms among Chinese middle-aged and older adults: evidence from two national cohorts
Source: BMC Psychiatry. 2024 Jun 19;24:458. doi: 10.1186/s12888-024-05909-7 (PMC11186271; doi:10.1186/s12888-024-05909-7)
Supplement: Supplementary file 1 — Supplementary Material 1 [file 12888_2024_5909_MOESM1_ESM.docx]

**Associations between eating alone, its transition and depressive symptoms among Chinese middle-aged and older adults: evidence from two national cohorts**

**Supplementary materials**

**1. The results of subgroup analysis of CFPS**

**Supplementary table1** subgroup analysis of association between eating alone and depressive symptoms in CFPS

| Subgroup | HR | SE | *P* | 95% CI | *P* for interaction |
| --- | --- | --- | --- | --- | --- |
| Age (years) |  |  |  |  |  |
| 45–59 | 1.268 | 0.167 | 0.072 | 0.979-1.641 |  |
| ≥60 | 1.491 | 0.197 | 0.002 | 1.151-1.930 | 0.694 |
| Sex |  |  |  |  |  |
| Male | 1.558 | 0.212 | 0.001 | 1.193-2.034 |  |
| Female | 1.212 | 0.154 | 0.130 | 0.945-1.555 | 0.069 |
| Residence |  |  |  |  |  |
| Urban residents | 1.285 | 0.207 | 0.119 | 0.937-1.762 |  |
| Rural residents | 1.414 | 0.160 | 0.002 | 1.134-1.765 | 0.304 |
| Marital status |  |  |  |  |  |
| Married | 1.465 | 0.182 | 0.002 | 1.147-1.870 |  |
| Unmarried | 1.232 | 0.168 | 0.126 | 0.943-1.608 | 0.534 |
| Educational level |  |  |  |  |  |
| Illiteracy | 1.296 | 0.187 | 0.072 | 0.977-1.720 |  |
| Primary school | 1.339 | 0.249 | 0.116 | 0.931-1.927 | 0.378 |
| Middle school and above | 1.440 | 0.233 | 0.024 | 1.049-1.978 | 0.416 |
| Income |  |  |  |  |  |
| Low | 1.216 | 0.161 | 0.140 | 0.938-1.576 |  |
| High | 1.509 | 0.197 | 0.002 | 1.168-1.948 | 0.104 |
| Smoking |  |  |  |  |  |
| No | 1.395 | 0.153 | 0.002 | 1.125-1.731 |  |
| Yes | 1.278 | 0.220 | 0.155 | 0.911-1.792 | 0.927 |
| Drinking |  |  |  |  |  |
| No | 1.290 | 0.133 | 0.013 | 1.055-1.579 |  |
| Yes | 1.853 | 0.382 | 0.003 | 1.237-2.775 | 0.113 |
| Exercise |  |  |  |  |  |
| No | 1.419 | 0.161 | 0.002 | 1.136-1.772 |  |
| Yes | 1.289 | 0.203 | 0.108 | 0.946-1.756 | 0.295 |
| Health insurance |  |  |  |  |  |
| No | 0.942 | 0.320 | 0.860 | 0.484-1.832 |  |
| Yes | 1.413 | 0.135 | <0.001 | 1.171-1.704 | 0.570 |
| Pension |  |  |  |  |  |
| No | 1.643 | 0.290 | 0.005 | 1.162-2.323 |  |
| Yes | 1.303 | 0.141 | 0.014 | 1.054-1.610 | 0.199 |
| Chronic disease |  |  |  |  |  |
| No | 1.417 | 0.150 | 0.001 | 1.152-1.744 |  |
| Yes | 1.252 | 0.233 | 0.227 | 0.869-1.804 | 0.388 |

**Supplementary table2** subgroup analysis of association between eating alone transition and depressive symptoms in CFPS

| Subgroup | Eating alone transition (Reference: commensality consistently) | HR | SE | *P* | 95% CI | *P* for interaction |
| --- | --- | --- | --- | --- | --- | --- |
| Age (years) |  |  |  |  |  |  |
| 45–59 | From eating alone to commensality | 1.217 | 0.218 | 0.272 | 0.857-1.728 |  |
|  | From commensality to alone | 1.493 | 0.205 | 0.003 | 1.141-1.953 |  |
|  | Eating alone consistently | 1.396 | 0.254 | 0.066 | 0.978-1.993 |  |
| ≥60 | From eating alone to commensality | 1.920 | 0.357 | <0.001 | 1.333-2.765 | 0.107 |
|  | From commensality to alone | 1.237 | 0.235 | 0.263 | 0.852-1.797 | 0.410 |
|  | Eating alone consistently | 1.315 | 0.214 | 0.093 | 0.956-1.809 | 0.491 |
| Sex |  |  |  |  |  |  |
| Male | From eating alone to commensality | 1.810 | 0.312 | 0.001 | 1.291-2.537 |  |
|  | From commensality to alone | 1.311 | 0.234 | 0.129 | 0.924-1.859 |  |
|  | Eating alone consistently | 1.385 | 0.253 | 0.075 | 0.968-1.982 |  |
| Female | From eating alone to commensality | 1.217 | 0.231 | 0.302 | 0.838-1.765 | 0.120 |
|  | From commensality to alone | 1.435 | 0.207 | 0.012 | 1.082-1.903 | 0.791 |
|  | Eating alone consistently | 1.258 | 0.205 | 0.158 | 0.915-1.732 | 0.292 |
| Residence |  |  |  |  |  |  |
| Urban residents | From eating alone to commensality | 1.314 | 0.309 | 0.246 | 0.829-2.085 |  |
|  | From commensality to alone | 1.650 | 0.337 | 0.014 | 1.106-2.462 |  |
|  | Eating alone consistently | 1.329 | 0.268 | 0.159 | 0.895-1.975 |  |
| Rural residents | From eating alone to commensality | 1.600 | 0.246 | 0.002 | 1.183-2.163 | 0.392 |
|  | From commensality to alone | 1.287 | 0.173 | 0.060 | 0.989-1.674 | 0.463 |
|  | Eating alone consistently | 1.307 | 0.200 | 0.080 | 0.968-1.764 | 0.554 |
| Marital status |  |  |  |  |  |  |
| Married | From eating alone to commensality | 1.468 | 0.221 | 0.011 | 1.094-1.971 |  |
|  | From commensality to alone | 1.361 | 0.176 | 0.017 | 1.056-1.755 |  |
|  | Eating alone consistently | 1.499 | 0.323 | 0.060 | 0.982-2.286 |  |
| Unmarried | From eating alone to commensality | 1.466 | 0.350 | 0.109 | 0.918-2.341 | 0.796 |
|  | From commensality to alone | 1.416 | 0.320 | 0.124 | 0.909-2.205 | 0.813 |
|  | Eating alone consistently | 1.235 | 0.186 | 0.161 | 0.919-1.659 | 0.594 |
| Educational level |  |  |  |  |  |  |
| Illiteracy | From eating alone to commensality | 1.542 | 0.333 | 0.045 | 1.009-2.354 |  |
|  | From commensality to alone | 1.219 | 0.217 | 0.266 | 0.860-1.727 | 0.437 |
|  | Eating alone consistently | 1.184 | 0.214 | 0.349 | 0.831-1.686 | 0.546 |
| Primary school | From eating alone to commensality | 1.780 | 0.382 | 0.007 | 1.169-2.711 |  |
|  | From commensality to alone | 1.285 | 0.289 | 0.266 | 0.826-1.998 | 0.686 |
|  | Eating alone consistently | 0.979 | 0.277 | 0.940 | 0.562-1.704 | 0.259 |
| Middle school and above | From eating alone to commensality | 1.230 | 0.294 | 0.387 | 0.770-1.966 |  |
|  | From commensality to alone | 1.673 | 0.312 | 0.006 | 1.161-2.412 | 0.876 |
|  | Eating alone consistently | 1.751 | 0.363 | 0.007 | 1.167-2.627 | 0.103 |
| Income |  |  |  |  |  |  |
| Low | From eating alone to commensality | 0.929 | 0.241 | 0.777 | 0.559-1.545 |  |
|  | From commensality to alone | 1.254 | 0.173 | 0.102 | 0.956-1.644 |  |
|  | Eating alone consistently | 1.401 | 0.216 | 0.029 | 1.035-1.896 |  |
| High | From eating alone to commensality | 1.858 | 0.278 | <0.001 | 1.386-2.491 | 0.114 |
|  | From commensality to alone | 1.674 | 0.324 | 0.008 | 1.145-2.447 | 0.189 |
|  | Eating alone consistently | 1.204 | 0.239 | 0.348 | 0.817-1.776 | 0.920 |
| Smoking |  |  |  |  |  |  |
| No | From eating alone to commensality | 1.464 | 0.230 | 0.015 | 1.076-1.994 |  |
|  | From commensality to alone | 1.346 | 0.178 | 0.025 | 1.038-1.745 |  |
|  | Eating alone consistently | 1.390 | 0.197 | 0.020 | 1.054-1.834 |  |
| Yes | From eating alone to commensality | 1.509 | 0.337 | 0.066 | 0.974-2.338 | 0.810 |
|  | From commensality to alone | 1.522 | 0.320 | 0.046 | 1.008-2.298 | 0.583 |
|  | Eating alone consistently | 1.126 | 0.267 | 0.616 | 0.708-1.793 | 0.931 |
| Drinking |  |  |  |  |  |  |
| No | From eating alone to commensality | 1.262 | 0.195 | 0.133 | 0.932-1.709 |  |
|  | From commensality to alone | 1.312 | 0.164 | 0.029 | 1.028-1.675 |  |
|  | Eating alone consistently | 1.351 | 0.176 | 0.021 | 1.047-1.744 |  |
| Yes | From eating alone to commensality | 2.537 | 0.553 | <0.001 | 1.654-3.890 | 0.118 |
|  | From commensality to alone | 1.762 | 0.455 | 0.028 | 1.062-2.922 | 0.254 |
|  | Eating alone consistently | 1.258 | 0.434 | 0.506 | 0.640-2.476 | 0.871 |
| Exercise |  |  |  |  |  |  |
| No | From eating alone to commensality | 1.565 | 0.240 | 0.003 | 1.159-2.113 |  |
|  | From commensality to alone | 1.171 | 0.171 | 0.279 | 0.880-1.558 |  |
|  | Eating alone consistently | 1.321 | 0.204 | 0.070 | 0.977-1.787 |  |
| Yes | From eating alone to commensality | 1.303 | 0.302 | 0.253 | 0.828-2.052 | 0.429 |
|  | From commensality to alone | 1.841 | 0.326 | 0.001 | 1.301-2.605 | 0.062 |
|  | Eating alone consistently | 1.385 | 0.273 | 0.098 | 0.942-2.038 | 0.576 |
| Health insurance |  |  |  |  |  |  |
| No | From eating alone to commensality | 1.319 | 0.709 | 0.607 | 0.459-3.785 |  |
|  | From commensality to alone | 2.432 | 1.150 | 0.060 | 0.962-6.146 |  |
|  | Eating alone consistently | 0.880 | 0.349 | 0.747 | 0.405-1.913 |  |
| Yes | From eating alone to commensality | 1.505 | 0.200 | 0.002 | 1.159-1.953 | 0.704 |
|  | From commensality to alone | 1.346 | 0.155 | 0.010 | 1.073-1.688 | 0.085 |
|  | Eating alone consistently | 1.383 | 0.175 | 0.011 | 1.079-1.772 | 0.714 |
| Pension |  |  |  |  |  |  |
| No | From eating alone to commensality | 1.731 | 0.437 | 0.030 | 1.055-2.839 |  |
|  | From commensality to alone | 1.486 | 0.350 | 0.092 | 0.937-2.357 |  |
|  | Eating alone consistently | 1.656 | 0.383 | 0.029 | 1.053-2.604 |  |
| Yes | From eating alone to commensality | 1.424 | 0.213 | 0.018 | 1.063-1.909 | 0.593 |
|  | From commensality to alone | 1.346 | 0.172 | 0.021 | 1.047-1.730 | 0.741 |
|  | Eating alone consistently | 1.245 | 0.177 | 0.124 | 0.942-1.646 | 0.199 |
| Chronic disease |  |  |  |  |  |  |
| No | From eating alone to commensality | 1.542 | 0.227 | 0.003 | 1.156-2.058 |  |
|  | From commensality to alone | 1.386 | 0.181 | 0.012 | 1.074-1.789 |  |
|  | Eating alone consistently | 1.371 | 0.191 | 0.024 | 1.043-1.803 |  |
| Yes | From eating alone to commensality | 1.325 | 0.344 | 0.278 | 0.797-2.202 | 0.585 |
|  | From commensality to alone | 1.377 | 0.305 | 0.149 | 0.892-2.125 | 0.861 |
|  | Eating alone consistently | 1.233 | 0.299 | 0.388 | 0.767-1.983 | 0.478 |

**2. The results of subgroup analysis of CHARLS**

**Supplementary table3** subgroup analysis of association between eating alone and depressive symptoms in CHARLS

| Subgroup | HR | SE | *P* | 95% CI | *P* for interaction |
| --- | --- | --- | --- | --- | --- |
| Age (years) |  |  |  |  |  |
| 45–59 | 1.167 | 0.112 | 0.109 | 0.966-1.409 |  |
| ≥60 | 1.191 | 0.108 | 0.054 | 0.997-1.424 | 0.696 |
| Sex |  |  |  |  |  |
| Male | 1.070 | 0.117 | 0.534 | 0.864-1.326 |  |
| Female | 1.229 | 0.103 | 0.013 | 1.044-1.447 | 0.604 |
| Residence |  |  |  |  |  |
| Urban residents | 0.964 | 0.136 | 0.796 | 0.731-1.271 |  |
| Rural residents | 1.251 | 0.094 | 0.003 | 1.080-1.449 | 0.124 |
| Marital status |  |  |  |  |  |
| Married | 1.113 | 0.099 | 0.231 | 0.934-1.325 |  |
| Unmarried | 1.280 | 0.135 | 0.019 | 1.041-1.575 | 0.380 |
| Educational level |  |  |  |  |  |
| Illiteracy | 1.084 | 0.141 | 0.535 | 0.840-1.397 |  |
| Primary school | 1.186 | 0.116 | 0.080 | 0.980-1.435 | 0.204 |
| Middle school and above | 1.225 | 0.149 | 0.097 | 0.964-1.555 | 0.249 |
| Income |  |  |  |  |  |
| Low | 1.185 | 0.093 | 0.031 | 1.016-1.383 |  |
| High | 1.119 | 0.135 | 0.352 | 0.883-1.419 | 0.970 |
| Smoking |  |  |  |  |  |
| No | 1.219 | 0.096 | 0.012 | 1.044-1.424 |  |
| Yes | 1.049 | 0.127 | 0.690 | 0.828-1.329 | 0.941 |
| Drinking |  |  |  |  |  |
| No | 1.198 | 0.093 | 0.020 | 1.029-1.396 |  |
| Yes | 1.102 | 0.138 | 0.436 | 0.863-1.408 | 0.672 |
| Exercise |  |  |  |  |  |
| No | 1.161 | 0.085 | 0.041 | 1.006-1.339 |  |
| Yes | 1.196 | 0.186 | 0.249 | 0.882-1.621 | 0.854 |
| Health insurance |  |  |  |  |  |
| No | 1.295 | 0.241 | 0.165 | 0.899-1.865 |  |
| Yes | 1.155 | 0.082 | 0.042 | 1.006-1.327 | 0.469 |
| Pension |  |  |  |  |  |
| No | 1.310 | 0.158 | 0.025 | 1.034-1.660 |  |
| Yes | 1.116 | 0.088 | 0.166 | 0.956-1.302 | 0.317 |
| Chronic disease |  |  |  |  |  |
| No | 1.158 | 0.130 | 0.191 | 0.930-1.442 |  |
| Yes | 1.174 | 0.096 | 0.050 | 1.000-1.378 | 0.995 |

**Supplementary table4** subgroup analysis of association between eating alone transition and depressive symptoms in CHARLS

| Subgroup | Eating alone transition (Reference: commensality consistently) | HR | SE | *P* | 95% CI | *P* for interaction |
| --- | --- | --- | --- | --- | --- | --- |
| Age (years) |  |  |  |  |  |  |
| 45–59 | From eating alone to commensality | 1.200 | 0.134 | 0.103 | 0.964-1.495 |  |
|  | From commensality to alone | 1.271 | 0.119 | 0.010 | 1.058-1.527 |  |
|  | Eating alone consistently | 1.174 | 0.197 | 0.338 | 0.845-1.631 |  |
| ≥60 | From eating alone to commensality | 1.026 | 0.147 | 0.855 | 0.775-1.359 | 0.260 |
|  | From commensality to alone | 1.267 | 0.122 | 0.014 | 1.048-1.531 | 0.791 |
|  | Eating alone consistently | 1.391 | 0.153 | 0.003 | 1.122-1.726 | 0.799 |
| Sex |  |  |  |  |  |  |
| Male | From eating alone to commensality | 0.990 | 0.143 | 0.943 | 0.745-1.314 |  |
|  | From commensality to alone | 1.334 | 0.137 | 0.005 | 1.091-1.630 |  |
|  | Eating alone consistently | 1.284 | 0.197 | 0.105 | 0.949-1.735 |  |
| Female | From eating alone to commensality | 1.214 | 0.136 | 0.083 | 0.975-1.511 | 0.289 |
|  | From commensality to alone | 1.206 | 0.107 | 0.035 | 1.014-1.434 | 0.421 |
|  | Eating alone consistently | 1.302 | 0.145 | 0.018 | 1.046-1.621 | 0.685 |
| Residence |  |  |  |  |  |  |
| Urban residents | From eating alone to commensality | 0.959 | 0.174 | 0.815 | 0.672-1.368 |  |
|  | From commensality to alone | 1.028 | 0.172 | 0.868 | 0.741-1.427 |  |
|  | Eating alone consistently | 0.978 | 0.201 | 0.914 | 0.654-1.463 |  |
| Rural residents | From eating alone to commensality | 1.189 | 0.121 | 0.089 | 0.974-1.451 | 0.343 |
|  | From commensality to alone | 1.331 | 0.098 | <0.001 | 1.153-1.537 | 0.199 |
|  | Eating alone consistently | 1.423 | 0.142 | <0.001 | 1.170-1.731 | 0.173 |
| Marital status |  |  |  |  |  |  |
| Married | From eating alone to commensality | 1.052 | 0.115 | 0.643 | 0.849-1.303 |  |
|  | From commensality to alone | 1.263 | 0.093 | 0.002 | 1.093-1.459 |  |
|  | Eating alone consistently | 1.322 | 0.195 | 0.059 | 0.989-1.766 |  |
| Unmarried | From eating alone to commensality | 1.306 | 0.209 | 0.095 | 0.955-1.787 | 0.271 |
|  | From commensality to alone | 1.319 | 0.217 | 0.093 | 0.955-1.821 | 0.901 |
|  | Eating alone consistently | 1.381 | 0.172 | 0.009 | 1.082-1.763 | 0.983 |
| Educational level |  |  |  |  |  |  |
| Illiteracy | From eating alone to commensality | 0.813 | 0.180 | 0.351 | 0.526-1.256 |  |
|  | From commensality to alone | 1.247 | 0.163 | 0.091 | 0.965-1.611 | 0.250 |
|  | Eating alone consistently | 1.345 | 0.206 | 0.053 | 0.996-1.816 | 0.127 |
| Primary school | From eating alone to commensality | 1.061 | 0.146 | 0.668 | 0.810-1.388 |  |
|  | From commensality to alone | 1.345 | 0.128 | 0.002 | 1.116-1.622 | 0.544 |
|  | Eating alone consistently | 1.444 | 0.182 | 0.004 | 1.128-1.849 | 0.684 |
| Middle school and above | From eating alone to commensality | 1.375 | 0.183 | 0.017 | 1.060-1.783 |  |
|  | From commensality to alone | 1.138 | 0.152 | 0.332 | 0.876-1.479 | 0.255 |
|  | Eating alone consistently | 0.951 | 0.228 | 0.836 | 0.595-1.522 | 0.430 |
| Income |  |  |  |  |  |  |
| Low | From eating alone to commensality | 1.136 | 0.115 | 0.209 | 0.931-1.386 |  |
|  | From commensality to alone | 1.308 | 0.110 | 0.001 | 1.109-1.543 |  |
|  | Eating alone consistently | 1.347 | 0.152 | 0.008 | 1.080-1.682 |  |
| High | From eating alone to commensality | 1.057 | 0.191 | 0.759 | 0.742-1.506 | 0.931 |
|  | From commensality to alone | 1.190 | 0.132 | 0.116 | 0.958-1.478 | 0.579 |
|  | Eating alone consistently | 1.214 | 0.179 | 0.188 | 0.910-1.620 | 0.938 |
| Smoking |  |  |  |  |  |  |
| No | From eating alone to commensality | 1.168 | 0.125 | 0.148 | 0.947-1.441 |  |
|  | From commensality to alone | 1.230 | 0.100 | 0.011 | 1.048-1.444 |  |
|  | Eating alone consistently | 1.345 | 0.144 | 0.006 | 1.091-1.658 |  |
| Yes | From eating alone to commensality | 1.022 | 0.161 | 0.890 | 0.750-1.393 | 0.756 |
|  | From commensality to alone | 1.338 | 0.157 | 0.013 | 1.063-1.684 | 0.400 |
|  | Eating alone consistently | 1.171 | 0.196 | 0.346 | 0.844-1.625 | 0.599 |
| Drinking |  |  |  |  |  |  |
| No | From eating alone to commensality | 1.190 | 0.124 | 0.093 | 0.971-1.459 |  |
|  | From commensality to alone | 1.396 | 0.108 | <0.001 | 1.200-1.624 |  |
|  | Eating alone consistently | 1.318 | 0.138 | 0.008 | 1.074-1.618 |  |
| Yes | From eating alone to commensality | 0.975 | 0.166 | 0.879 | 0.698-1.361 | 0.345 |
|  | From commensality to alone | 1.012 | 0.137 | 0.929 | 0.777-1.319 | 0.041 |
|  | Eating alone consistently | 1.280 | 0.225 | 0.160 | 0.907-1.805 | 0.908 |
| Exercise |  |  |  |  |  |  |
| No | From eating alone to commensality | 1.100 | 0.107 | 0.325 | 0.909-1.332 |  |
|  | From commensality to alone | 1.305 | 0.095 | <0.001 | 1.131-1.506 |  |
|  | Eating alone consistently | 1.317 | 0.132 | 0.006 | 1.083-1.602 |  |
| Yes | From eating alone to commensality | 1.201 | 0.255 | 0.388 | 0.792-1.821 | 0.902 |
|  | From commensality to alone | 1.113 | 0.191 | 0.532 | 0.795-1.559 | 0.285 |
|  | Eating alone consistently | 1.226 | 0.258 | 0.333 | 0.812-1.851 | 0.609 |
| Health insurance |  |  |  |  |  |  |
| No | From eating alone to commensality | 1.132 | 0.320 | 0.661 | 0.650-1.971 |  |
|  | From commensality to alone | 1.522 | 0.284 | 0.025 | 1.055-2.194 |  |
|  | Eating alone consistently | 1.561 | 0.366 | 0.058 | 0.985-2.472 |  |
| Yes | From eating alone to commensality | 1.121 | 0.105 | 0.222 | 0.933-1.346 | 0.932 |
|  | From commensality to alone | 1.237 | 0.089 | 0.003 | 1.074-1.424 | 0.295 |
|  | Eating alone consistently | 1.265 | 0.124 | 0.016 | 1.045-1.532 | 0.270 |
| Pension |  |  |  |  |  |  |
| No | From eating alone to commensality | 1.069 | 0.192 | 0.710 | 0.752-1.520 |  |
|  | From commensality to alone | 1.370 | 0.181 | 0.017 | 1.057-1.774 |  |
|  | Eating alone consistently | 1.720 | 0.271 | 0.001 | 1.262-2.343 |  |
| Yes | From eating alone to commensality | 1.127 | 0.115 | 0.240 | 0.923-1.377 | 0.686 |
|  | From commensality to alone | 1.229 | 0.096 | 0.008 | 1.055-1.431 | 0.559 |
|  | Eating alone consistently | 1.167 | 0.128 | 0.160 | 0.941-1.448 | 0.070 |
| Chronic disease |  |  |  |  |  |  |
| No | From eating alone to commensality | 1.056 | 0.161 | 0.722 | 0.783-1.424 |  |
|  | From commensality to alone | 1.283 | 0.151 | 0.034 | 1.018-1.616 |  |
|  | Eating alone consistently | 1.384 | 0.216 | 0.038 | 1.019-1.88 |  |
| Yes | From eating alone to commensality | 1.151 | 0.125 | 0.197 | 0.930-1.425 | 0.664 |
|  | From commensality to alone | 1.255 | 0.102 | 0.005 | 1.070-1.472 | 0.873 |
|  | Eating alone consistently | 1.271 | 0.140 | 0.029 | 1.025-1.576 | 0.633 |
